# The impact of access to sanitary toilets on rural adult residents' health: Evidence from the China family panel survey

**DOI:** 10.3389/fpubh.2022.1026714

**Published:** 2022-12-09

**Authors:** Baoqi Chen, Fulei Jin, Yaxin Zhu

**Affiliations:** School of Economics, Shandong University of Finance and Economics, Jinan, China

**Keywords:** toilet revolution, rural residents' health, protection of water sources, ordered probit (OP) model, mechanism testing, heterogeneity

## Abstract

Toilet sanitation is related to public health and environmental protection. In the context of the toilet revolution in rural China, an ordered probit regression analysis was conducted to evaluate the impact of access to sanitary toilets on rural residents' health. Using data from the China Family Panel Study (CFPS) in 2014, we found that access to sanitary toilets in rural households significantly improved residents' health, and this finding remained robust across a series of checks. Meanwhile, results of the mechanism analysis showed that preventing feces from contaminating water sources was an important mechanism behind the positive effects of sanitary toilet use on health. We also found that the impact of access to sanitary toilets was more pronounced among female, middle-aged, and low-income people. Toilet revolution plays an important role in ensuring residents' health and protecting water sources, thereby underscoring the need for governments in developing countries to invest in sanitary toilets. In addition, the existing policies and sanitation programs in China need to be improved to promote public health.

## Introduction

With the development of society, toilet sanitation has been regarded as an important factor affecting social progress and even considered the barometer of civilization (1). According to the United Nations International Children's Emergency Fund (UNICEF) and the World Health Organization joint report, as of 2015, 2.4 million people still did not have access to sanitary toilets in the world, and 946 million people still practiced open defection. Even in urban areas where private and public toilets are more prevalent, over 2 billion people were using unsanitary toilet facilities that discharged raw sewage into open drains or surface waters. The public health problems caused by poor toilet facilities have been a common concern of governments and academics in developing countries.

Unfortunately, due to the unbalanced development between urban and rural areas (2, 3), the situation in rural China is not as positive. Specifically, the imbalanced socioeconomic development has resulted in the variability of rural infrastructure penetration (4), thereby forcing many residents in the vast rural areas of China to continue using collective dry toilets. According to official data, as of 2016, the coverage rate of sanitary toilets in rural areas was only 36.2%, while 12.4% of farmers used unsanitary toilets and 2% still had no access to toilets. Poor toilet facilities expose 17 million households to diseases and infections every year (5). In response to this issue, Chinese Prime Minister Xi Jinping said that China would launch a “toilet revolution” to let rural residents use sanitary toilets. This revolution is tightly associated with the patriotic health campaign in China, which started in the 1950s with an aim to improve sanitation and eliminate diseases (6). Since 2004, China has allocated RMB 8.64 billion to the renovation of rural toilets. The goal of the toilet revolution is to reach 85 and 100% coverage rates of sanitary toilets by 2020 and 2030, respectively.

The relationship between toilet sanitation and public health has attracted much attention in recent years. According to medical and epidemiological studies, the lack of sanitary toilets exacerbates the spread of viruses, such as Escherichia coli, Salmonella, and other pathogens, thereby increasing the incidence of worms, schistosomiasis, malaria, diarrhea, and other diseases (7–9). In addition, the frequency of diseases caused by fecal pathogens is closely related to malnutrition, growth stagnation, underweight, and short height (10–18). In particular, the spread of diarrhea attributable to the poor coverage of sanitary toilets causes 1.5 million deaths among children every year (19), making this disease the second leading cause of morbidity among children under 5 years and the main cause of deaths in sub-Saharan Africa (20–22). From the environmental protection perspective, other scholars found that poor toilet facilities increase the risk of fecal contamination on soil and water sources (23, 24), especially in low-income developing countries (25–27). From the social welfare perspective, several studies have analyzed the impact of investment in toilets on poverty and well-being. For example, Yang et al. (28) found that low net income per capital, low levels of education, and low penetration of sanitary toilets are the main causes of poverty in rural China. Gonsalves et al. (29) found that increasing the coverage of toilets in rural areas can reduce the incidence of sexual assaults and greatly improve the security of women. Ao et al. (30) focused on farmers' satisfaction with infrastructure construction and found that public toilet renovation can significantly improve their satisfaction.

Despite the significance of the toilet revolution and the efforts of China's government, only few studies have examined the nexus between toilet revolution and rural adult residents' health at the micro-individual level. Using China Family Panel Studies (CFPS) data, this work offers several contributions to the literature. First, using excellent nationwide micro-survey data from CFPS allows us to control individual, household, and village characteristics and improve the accuracy of the quantitative analysis. Second, the ordered probit model is employed to examine how access to sanitary toilets can affect residents' health in rural China, and a series of checks is performed to test the robustness of the results. Third, the mechanism behind the protective effect of access to sanitary toilets is tested, and the heterogenous impact on different groups is evaluated.

The rest of this paper is organized as follows. Materials and methods introduces the data, definitions of terms, and adopted empirical strategy. Results presents the results of empirical analysis. Discussion outlines the discussion, and Conclusions concludes the paper.

## Materials and methods

### Data source

The data were derived from CFPS, a social survey developed and administered by the Institute of Social Science Survey (ISSS) of Peking University. The CFPS baseline survey was officially conducted in 2010, and the data were collected *via* interviews every 2 years. Six waves of this survey were conducted from 2010 to 2020, which covered 25 provinces with 16,000 households and included all household members. However, the response categories for self-rated health in 2010 were not consistent with those in later waves because of changes in the questionnaire design. Moreover, the village survey was not conducted in 2012, and the toilet survey was not conducted in 2016, 2018, and 2020. Therefore, the analysis in this paper was confined to the third wave of the CFPS (2014), whose data is suitable for this study.

In this paper, the relationship between toilet revolution and rural residents' health was investigated. The CPFS in 2014 involved 37,451 respondents, and the analytical sample was derived through the following steps. First, those respondents whose villages were not included in the village survey were dropped (4,500). Second, given that this study focused on residents' health in rural areas, those individuals living in urban areas were excluded (7694). Third, given that those individuals aged 16 years and below were defined as adolescent in the CFPS, these individuals were excluded (342). Fourth, those respondents who reported missing data on individual-, household-, or village-level explanatory variables were excluded (6262). A total of 18,650 valid responses, which covered 381 villages and 7,600 households, were eventually obtained after the data screening.

#### Outcome variable

The key outcome variable of this study was self-rated health, an indicator of overall health status that has been utilized in many social surveys (31–34). Specifically, self-rated health was measured by asking the respondents to evaluate their overall health status from 1 to 5, where 1 represented “poor” and 5 represented “excellent.”

#### Focal variable

Collective dry toilets are still popular in rural areas in China, whereas flush toilets, which the governments aims to install in these areas, are considered prestigious and desirable (5). In this survey, each interviewee was asked to choose the type of toilet s/he used the most. Specifically, they were asked to choose among indoor flush toilet, outdoor private flush toilet, outdoor public flush toilet, indoor non-flush toilet, outdoor private non-flush toilet, and outdoor public non-flush toilet. The variable “sanitary toilet” was equal to 1 if the respondent used flush toilet and equal to 0 if s/he used non-flush toilet.

#### Control variables

Other variables that may affect both the outcome and focal variables were also included in the analysis, such as individual characteristics (e.g., gender, age, marital status, and faith) and individual behaviors (e.g., smoking, internet use, reading, alcoholism, and noon breaks). To crowd out other confounding factors, the data for individuals, families, and villages were matched. Household characteristics included net household income per capita, family size, and water, whereas village characteristics included a series of variables representing the living status of respondents (e.g., whether the village was located near highly polluting enterprises, whether the village had hospitals, and the distance from the village committee to the county capital). The regional heterogeneity was controlled by a series of village dummy variables.

The variable selection and sample characteristics are shown in Table 1.

**Table 1:** Variable definitions and descriptive statistics.

**Variable and value**	**Definition**	***N*/Mean**	**%Std**
**Outcome variable**			
Self-rated health	How would you rate your health status?		
1	Poor	3,319	17.80
2	Fair	2,635	14.13
3	Good	6,003	32.19
4	Very good	3,888	20.85
5	Excellent	2,805	15.04
**Focal variable**			
Sanitary toilet	What kind of restroom/toilet facilities does your family use mostly?		
0	Indoor non-flush toilet, outdoor private non-flush toilet, or outdoor public non-flush toilet	13,189	70.72
1	Indoor flush toilet, outdoor private flush toilet, or outdoor public flush toilet	5,461	29.28
**Control variables**			
Gender	Gender of respondents		
0	Female	9,414	50.48
1	Male	9236	49.52
Age	Age of respondents	46.72826	16.42719
Marital status	What is your marital status?		
0	Not in a relationship	3,447	18.48
1	In a relationship	15,203	81.52
Education	What is your education level?		
1	Illiterate	6,487	34.78
2	Primary school	4,591	24.62
3	Junior middle school	5,140	27.56
4	High school	1,825	9.79
5	Junior college	407	2.18
6	College and above	200	1.07
Noon break	Do you take a noon break?		
0	No	9,260	49.65
1	Yes	9,390	50.35
Smoking	Did you smoke cigarettes in the past month?		
0	No	13,054	69.99
1	Yes	5,596	30.01
Alcoholism	Did you drink alcohol at least 3 times a week in the past month?		
0	No	15,670	84.02
1	Yes	2,980	15.98
Reading	Have you read any books in the past year for purposes other than work or exams?		
0	No	15,309	82.09
1	Yes	33,41	17.91
Exercise	How often did you participate in physical exercise in the past week?		
0	0	13,274	71.17
1	≥1	5376	28.83
Internet use	Do you use Internet?		
0	No	14,589	78.23
1	Yes	4,061	21.77
Faith	Do you have any religious beliefs?		
0	No	13,511	72.45
1	Yes	5,139	27.55
Family size	Number of people in the family	4.578123	2.020893
Family income	Logarithm of per capital income of the household	8.803186	1.206061
Water	What kind of water does your family use for cooking?		
0	River and lake water, well water, rainwater, cistern water, or pool water	8,278	44.39
1	Tap water, bottled water, pure water, or filtered water	10,372	55.61
Playground	Is there a playground in your village?		
0	No	9,524	51.07
1	Yes	9,126	48.93
Drug store	Is there a drug store in your village?		
0	No	11,563	62
1	Yes	7,087	38
Hospital	Is there a hospital in your village?		
0	No	2,981	15.98
1	Yes	15,669	84.02
Highly polluting enterprise	Is there a highly polluting enterprise in your village?		
0	No	15,508	83.15
1	Yes	3,142	16.85
Ethnic minority area	Is your village an ethnic minority area?		
0	No	16,600	89.01
1	Yes	2,050	10.99
Distance	Distance between your village committee office and the county capital	48.63051	39.76809
Village size	Logarithm of the number of people in the family	7.540707	0.7729587

### Econometric model

We examined the impact of toilet revolution on the health status of adults among rural households using an ordered probit model (oprobit). Unlike linear regression models, the oprobit model can fit non-linear models by dealing with situations where the outcome “health” is an ordered variable (35, 36). The regression model in this study is expressed as


(1) $$\begin{array}{l} {Health}_{i}=F\left(\alpha +\beta Toilet_{i}+\sum_{m}X^{m}+\mu_{i}\right) \end{array}$$

where the outcome variable (Health) stands for the health of individual i, the key explanatory variable (Toilet) indicates whether the respondent uses a sanitary toilet, X represents a series of control variables, μ is a random disturbance term, and the function F is defined as


(2) $$\begin{array}{l} \text{F}\left(Health^{*}\right)=\{\begin{array}{cc} 1,\; & if\;health^{*}<\mu_{1}\\ 2,\; & if\;\mu_{1}{<health}^{*}<\mu_{2}\\ J,\; & \;if\;\mu_{j-1}<health^{*}<\mu_{j} \end{array} \end{array}$$

Where Health^*^ is the latent variable of Health, which satisfies the following equation:


(3) $$\begin{array}{l} {Health}_{i}^{*}=\alpha +\beta Toilet_{i}+\sum_{m}X^{m}+\mu_{i} \end{array}$$

## Results

### The baseline estimation results

The results of the oprobit model are shown in columns (1) and (2) of Table 2. Only the focal variable was controlled in column (1), which shows that the parameter of the focal variable is 0.179, which is significant at the 1% level. In column (2) where the control variables were added, sanitary toilet (0.066) has a statistically significant impact on self-rated health at the 5% level, gender (0.177), age (−0.024), marital status (−0.059), education (0.050), noon break (−0.079), smoking (0.064), alcoholism (0.217), exercise (0.110), family size (0.023), and net household income per capita (0.038) have statistically significant impacts on self-rated health at the 1% level, and faith (−0.049) and village size (−0.423) have significant effects on self-rated health at the 5% level.

**Table 2:** Results of the baseline estimation and robustness check.

**Variables**	**(1)**	**(2)**	**(3)**	**(4)**	**(5)**	**(6)**
	**Self–rated health**	**Self–rated health**	**Interviewer–rated health**	**Interviewer–rated health**	**Life satisfaction**	**Life satisfaction**
Sanitary toilet	0.179^***^	0.066^**^	0.241^***^	0.095^***^	0.190^***^	0.178^***^
	(0.0260)	(0.0265)	(0.0263)	(0.0270)	(0.0266)	(0.0269)
Gender		0.177^***^		0.043^**^		−0.031
		(0.0208)		(0.0211)		(0.0210)
Age		−0.024^***^		−0.021^***^		0.004^***^
		(0.0007)		(0.0007)		(0.0007)
Marital status		−0.059^***^		0.134^***^		0.123^***^
		(0.0221)		(0.0224)		(0.0222)
Education		0.050^***^		0.119^***^		−0.020^**^
		(0.0090)		(0.0093)		(0.0091)
Noon break		−0.079^***^		−0.008		0.062^***^
		(0.0172)		(0.0176)		(0.0174)
Smoking		0.064^***^		0.045^**^		−0.013
		(0.0221)		(0.0226)		(0.0224)
Alcoholism		0.217^***^		0.153^***^		0.028
		(0.0243)		(0.0249)		(0.0246)
Reading		−0.007		0.155^***^		0.028
		(0.0241)		(0.0251)		(0.0245)
Exercise		0.110^***^		0.094^***^		0.170^***^
		(0.0189)		(0.0193)		(0.0192)
Internet use		0.002		0.076^***^		0.036
		(0.0264)		(0.0273)		(0.0267)
Faith		−0.049^**^		−0.026		−0.014
		(0.0201)		(0.0204)		(0.0203)
Family size		0.023^***^		0.015^***^		0.021^***^
		(0.0046)		(0.0047)		(0.0047)
Family income		0.038^***^		0.045^***^		0.059^***^
		(0.0075)		(0.0075)		(0.0075)
Water		−0.013		0.061^**^		−0.005
		(0.0240)		(0.0243)		(0.0243)
Playground		0.315		3.038^***^		−0.314
		(0.3502)		(0.3745)		(0.3634)
Drug store		−0.216		−9.063^***^		−0.225
		(0.8389)		(0.9541)		(0.8763)
Hospital		0.006		−13.790^***^		−0.348
		(1.3956)		(1.5543)		(1.4519)
Polluting enterprise		−0.424		−5.580^***^		−0.625
		(0.4892)		(0.5465)		(0.5072)
Ethnic minority area		−0.055		12.516^***^		0.978
		(1.2451)		(1.4137)		(1.2994)
Distance		−0.016		−0.182^***^		−0.015
		(0.0172)		(0.0195)		(0.0179)
Village size		−0.423^**^		0.856^***^		−0.091
		(0.1928)		(0.2053)		(0.1956)
Village fixed effects	Yes	Yes	Yes	Yes	Yes	Yes
Obs	18650	18650	18646	18646	18627	18627

*** p < 0.01,

** p < 0.05,

*p < 0.1.

### Analysis of marginal effects

Given that the oprobit regression is a non-linear model, the information derived from the parameters and significance is limited in Table 2. To obtain the results intuitively, the marginal effects of access to sanitary toilets on health status are shown in Table 3. The likelihoods of “poor,” “fair,” and “good” self-rated health decrease by 1.46, 0.55, and 0.07%, respectively, whereas the likelihoods of “very good” and “excellent” self-rated health increase by 0.78 and 1.3%, respectively, at the 5% level.

**Table 3:** Marginal effects of access to sanitary toilets on health.

**Variables**	**Poor**	**Fair**	**Good**	**Very good**	**Excellent**
Sanitary toilet	−0.0149^**^ (0.0059)	−0.0056^**^ (0.0022)	−0.0007^**^ (0.0003)	0.0079^**^ (0.0031)	0.0130^**^ (0.0053)
Control variable	Yes	Yes	Yes	Yes	Yes
Village fixed effects	Yes	Yes	Yes	Yes	Yes
Obs	18,650	18,650	18,650	18,650	18,650

***p < 0.01,

** p < 0.05,

*p < 0.1.

In sum, residents' health can be improved by using sanitary toilets. Specifically, the use of sanitary toilets can significantly increase the likelihood of obtaining “very good” and “excellent” self-rated health and reduce the likelihood of obtaining “poor,” “fair,” and “good” self-rated health.

### Robustness check

#### Replacement of health indicators

In the following, self-rated health was replaced with two count variables, namely, interviewer-rated health and life satisfaction (Table 4). The first measure, interviewer-rated health, is also an indicator of overall health. After each interview, the interviewer was asked to evaluate the health status of the respondents and select one of seven categories, which ranged from 1 (very poor) to 7 (very excellent). To be more objective and accurate compared with self-rated health, the interviewer-rated health was also used as an outcome variable. The second measure, life satisfaction, is an indicator of subjective well-being and reflects the respondents' assessments of health status to some extent. Specifically, life satisfaction was measured on 5-point Likert scale using the question, “How satisfied are you with your life?” To maximize statistical power, the sample size varied depending on the number of valid observations for each outcome variable. Consequently, the sample sizes ranged from 18,627 for life satisfaction to 18,646 for interviewer-rated heath.

**Table 4:** Variable definitions and descriptive statistics.

**Variable and value**	**Definition**	***N***	**%**
Interviewer–rated health	Respondent's health status		
1	Very poor	108	0.58
2	Poor	293	1.57
3	Fair	880	4.72
4	Good	2,385	12.80
5	Very good	4,926	26.42
6	Excellent	6,233	33.43
7	Very excellent	3,820	20.49
Life satisfaction	Are you satisfied with your life?		
1	Very unsatisfied	510	2.74
2	Unsatisfied	1077	5.78
3	Fair	5383	28.90
4	Satisfied	6007	32.25
5	Very satisfied	5650	30.33

Columns (3) to (6) of Table 2 present the effects of access to sanitary toilets on interviewer-rated health and life satisfaction as estimated by the oprobit model. As shown in column (4) of Table 2, the parameter of sanitary toilet is 0.095, which is significant at 1% level, thereby highlighting the positive effect of using sanitary toilets on interviewer-rated health. Moreover, age (−0.021), marital status (0.134), education (0.119), alcoholism (0.153), reading (0.155), exercise (0.094), family size (0.015), net household income per capita (0.045), playground (3.038), drug store (−9.063), hospital (−13.790), polluting enterprise (−5.580), ethnic minority area (12.516), distance (−0.182), and village size (0.856) have statistically significant impacts on interviewer-rated health at the 1% level, whereas gender (0.043), and smoking (0.045) significantly affect interviewer-rated health at the 5% level.

Column (6) of Table 2 show that the influence coefficient of the key explanatory variable (sanitary toilet) is 0.178, which is significant at the 1% level, thereby highlighting a positive association between access to sanitary toilets and life satisfaction. Additionally, age (0.004), marital status (0.123), education (−0.020), noon break (0.062), exercise (0.170), family size (0.021), and net household income per capita (0.059) have statistically significant impacts on life satisfaction.

By replacing the health indicators, the positive effect of access to sanitary toilet on health was proven robust.

#### Alternate sample

Physically disabled residents usually assess their self-rated health as “poor.” However, the disabled residents prefer to use sanitary toilets, which may cause selective bias and inconsistent estimates. In the CFPS, the respondents aged over 45 years were asked, “Which of the following activities can you not perform independently?” On the basis of the responses to this question, the disabled respondents were removed from the sample of residents over 45 years, and another estimation was performed. As shown in Table 5, the parameters of sanitary toilet are 0.095, 0.133, and 0.161, which are all significant at the 1% level, thereby confirming the robustness of the estimations.

**Table 5:** Robustness check by alternate sample.

**Variables**	**(1)**	**(2)**	**(3)**
	**Self–rated health**	**Interviewer–rated health**	**Life satisfaction**
Sanitary toilet	0.095^**^	0.133^***^	0.161^***^
	(0.0381)	(0.0384)	(0.0389)
Control variable	Yes	Yes	Yes
Village fixed effects	Yes	Yes	Yes
Obs	9,478	9,475	9,462

*** p < 0.01,

** p < 0.05,

*p < 0.1.

### Mechanism analysis

This part explores the mechanism by which access to sanitary toilets can impact health status. Water safety has an important influence on human health (37), and many diseases, such as diarrhea, are caused by fecal pollution of water sources (e.g., rivers, lakes, ponds, and wells) (38–41). Given that using sanitary toilets at the dwelling can prevent feces from contaminating water sources and thus reduce the incidence of waterborne diseases, several scholars argued that protecting water sources is the mechanism behind the effect of sanitary toilets on public health (24, 42).

In China, the aforementioned mechanism is closely related to the rural drinking water safety project, an intervention policy aimed toward enhancing the quality of drinking water in rural China. One goal of this project was to establish piped water supply systems. When a household is connected to a tap water system, the water used is taken directly from natural water sources and passes through a series of treatment processes (43). The positive effect of using tap water on health has been proven in a series of studies (44–48). Therefore, the likelihood for family members from tap-water-drinking households to catch diseases from drinking unsafe drinking water is greatly reduced even they do not have a sanitary toilet. This analysis makes full use of the rural drinking water safety project to test whether protecting water sources drives the effect of sanitary toilets on public health.

The sample was divided into the “tap water” and “no tap water” groups, and the latter was regarded as the reference group. The oprobit model was adopted in the analysis. If the protective effect of sanitary toilet on health is achieved by protecting water sources, then using sanitary toilets will only have a limited effect on the health of residents whose dwellings are connected to piped water. In other words, the parameter of the focal variable (sanitary toilet) would be either small or not significant in the “tap water” group yet significant in the “no tap water” group. Conversely, if the protective effect of sanitary toilets on health is not achieved by protecting water sources, then the regression parameter (sanitary toilet) would remain significant even in the “tap water” group.

Columns (1) and (2) of Table 6 show that the parameter of sanitary toilet in the “no tap water” group is 0.092, which is significant at the 5% level, whereas that in the “tap water” group is 0.039, which fails the statistical test. Columns (3) and (4) use interviewer-rated health as the outcome variable to estimate again, and the results are consistent with those obtained using self-rated health. These findings suggest that preventing fecal pathogens from contaminating water sources is an important mechanism by which access to sanitary toilets can improve residents' health.

**Table 6:** Mechanism analysis.

**Variables**	**(1)**	**(2)**	**(3)**	**(4)**
	**No tap water**	**Tap water**	**No tap water**	**Tap water**
	**Self–rated health**	**Self–rated health**	**Interviewer–rated health**	**Interviewer–rated health**
Sanitary toilet	0.092^**^	0.039	0.176^***^	0.057
Control variable	Yes	Yes	Yes	Yes
Village fixed effects	Yes	Yes	Yes	Yes
Obs	7,391	10,372	7,389	10,370

*** p < 0.01,

** p < 0.05,

*p < 0.1.

### Heterogeneity

#### Gender

The physiological differences between males and females have resulted in the weak position of the latter in rural China. This part tests for any heterogeneity in the effects of the toilet revolution on self-rated health in terms of gender. The sample was then divided into females (9,414) and males (9,236).

The results in Table 7 show that access to sanitary toilets significantly improves the health of females (0.070) and males (0.064). Moreover, marital status (−0.076), faith (−0.073), and village size (−0.570) significantly affect females' health but have no significant impact on that of males. Age, education, noon break, smoking, alcoholism, exercise, family size, and net household income per capita all have significant impacts on the health of females (−0.024, 0.061, −0.069, −0.130, 0.178, 0.138, 0.026, and 0.028, respectively) and males (−0.025, 0.038, −0.091, 0.073, 0.230, 0.085, 0.020, and 0.049, respectively).

**Table 7:** Heterogeneity analysis of gender, age and income.

**Variables**	**Female**	**Male**	**Young**	**Middle–aged**	**Older**	**Low–income**	**Middle–income**	**High–income**
	**Self–reported health**	**Self–reported health**	**Self–reported health**	**Self–reported health**	**Self–rated health**	**Self–reported health**	**Self–reported health**	**Self–rated health**
Sanitary toilet	0.070^*^	0.064^*^	0.067^*^	0.089^*^	0.082	0.112^**^	0.066	0.022
	(0.0379)	(0.0376)	(0.0403)	(0.0496)	(0.0582)	(0.0541)	(0.0505)	(0.0449)
Gender			0.198^***^	0.218^***^	0.086^**^	0.113^***^	0.245^***^	0.172^***^
			(0.0318)	(0.0401)	(0.0433)	(0.0365)	(0.0370)	(0.0367)
Age	−0.024^***^	−0.025^***^	−0.028^***^	−0.033^***^	−0.007^**^	−0.025^***^	−0.027^***^	−0.023^***^
	(0.0010)	(0.0010)	(0.0021)	(0.0035)	(0.0028)	(0.0011)	(0.0012)	(0.0013)
Marital status	−0.076^**^	−0.034	−0.082^**^	0.127^*^	0.04	−0.044	−0.052	−0.076^*^
	(0.0317)	(0.0326)	(0.0368)	(0.0659)	(0.0455)	(0.0391)	(0.0403)	(0.0395)
Education	0.061^***^	0.038^***^	0.077^***^	0.050^***^	0.001	0.068^***^	0.067^***^	0.026^*^
	(0.0137)	(0.0125)	(0.0137)	(0.0162)	(0.0232)	(0.0179)	(0.0162)	(0.0147)
Noon break	−0.069^***^	−0.091^***^	−0.048^*^	−0.059^*^	−0.180^***^	−0.069^**^	−0.093^***^	−0.078^***^
	(0.0245)	(0.0249)	(0.0265)	(0.0320)	(0.0372)	(0.0316)	(0.0309)	(0.0300)
Smoking	−0.130^*^	0.073^***^	0.046	0.100^**^	0.119^***^	0.064	0.081^**^	0.104^***^
	(0.0701)	(0.0242)	(0.0357)	(0.0407)	(0.0438)	(0.0396)	(0.0395)	(0.0391)
Alcoholism	0.178^**^	0.230^***^	0.147^***^	0.227^***^	0.342^***^	0.263^***^	0.248^***^	0.144^***^
	(0.0708)	(0.0269)	(0.0399)	(0.0423)	(0.0494)	(0.0449)	(0.0441)	(0.0407)
Reading	−0.023	0.005	−0.084^***^	0.047	0.188^***^	0.005	−0.052	−0.001
	(0.0386)	(0.0317)	(0.0313)	(0.0520)	(0.0696)	(0.0483)	(0.0426)	(0.0394)
Exercise	0.138^***^	0.085^***^	0.112^***^	0.009	0.218^***^	0.107^***^	0.110^***^	0.125^***^
	(0.0272)	(0.0269)	(0.0295)	(0.0357)	(0.0391)	(0.0354)	(0.0340)	(0.0321)
Internet use	0.023	−0.015	0.011	−0.08	−0.231	0.044	−0.019	0.024
	(0.0391)	(0.0365)	(0.0330)	(0.0709)	(0.2240)	(0.0522)	(0.0464)	(0.0443)
Faith	−0.073^***^	−0.026	−0.069^**^	−0.042	−0.05	−0.045	−0.014	−0.082^**^
	(0.0280)	(0.0300)	(0.0312)	(0.0374)	(0.0427)	(0.0365)	(0.0364)	(0.0352)
Family size	0.026^***^	0.020^***^	0.028^***^	0.016^*^	0.019^**^	0.024^***^	0.022^**^	0.021^**^
	(0.0066)	(0.0066)	(0.0077)	(0.0090)	(0.0091)	(0.0083)	(0.0086)	(0.0097)
Family income	0.028^***^	0.049^***^	−0.001	0.085^***^	0.063^***^	−0.012	0.152^**^	0.071^**^
	(0.0106)	(0.0107)	(0.0116)	(0.0142)	(0.0161)	(0.0143)	(0.0631)	(0.0351)
Water	0.009	−0.034	−0.002	−0.019	−0.051	−0.026	0.029	−0.038
	(0.0340)	(0.0342)	(0.0364)	(0.0445)	(0.0526)	(0.0435)	(0.0446)	(0.0456)
Playground	0.741	−0.02	0.553	0.69	−0.155	1.016	0.692	0.077
	(0.4971)	(0.4970)	(0.6470)	(0.5660)	(0.6349)	(1.0156)	(0.6466)	(0.5531)
Drug store	−0.893	0.28	−0.978	−0.904	0.797	−1.914	−1.052	0.407
	(1.1633)	(1.2211)	(1.6301)	(1.3524)	(1.4823)	(2.0182)	(1.7881)	(1.4436)
Hospital	−0.889	0.695	−1.995	−0.007	1.618	−3.38	−0.301	0.676
	(1.9480)	(2.0162)	(2.6379)	(2.2521)	(2.5802)	(3.5069)	(2.8147)	(2.4581)
Polluting enterprise	−0.939	−0.016	−1.025	−0.622	0.192	−1.142	−0.798	−0.396
	(0.6822)	(0.7079)	(0.9208)	(0.7826)	(0.9317)	(1.1678)	(0.9852)	(0.8731)
Ethnic minority area	0.886	−0.736	0.836	1.216	−0.869	2.153	0.674	−0.212
	(1.7276)	(1.8115)	(2.4132)	(1.9936)	(2.2443)	(2.9839)	(2.6581)	(2.1653)
Distance	−0.032	−0.004	−0.023	−0.043	−0.001	−0.04	−0.04	−0.011
	(0.0239)	(0.0249)	(0.0332)	(0.0275)	(0.0309)	(0.0413)	(0.0360)	(0.0302)
Village size	−0.570^**^	−0.306	0.049	−0.747^**^	−0.699^*^	0.169	−0.594^*^	−0.608
	(0.2752)	(0.2721)	(0.3311)	(0.3287)	(0.3945)	(0.4309)	(0.3417)	(0.3802)
Village fixed effects	Yes	Yes	Yes	Yes	Yes	Yes	Yes	Yes
Obs	9,414	9,236	8,130	5,860	4,660	6,216	6,246	6,241

*** p < 0.01,

** p < 0.05,

* p < 0.1.

#### Age

Individuals experience different physical and psychological conditions across their life stages. Therefore, the heterogeneity in the influence of the toilet revolution on health in terms of age cannot be ignored. Following the United Nations' standards, those individuals aged between 17 and 44 years were classified as the young group (8,130), those aged 45 to 59 years were classified as the middle-aged group (5,840), and those aged over 60 years were classified as the older group (4,660).

As shown in Table 7, access to sanitary toilets has a significant impact on the health of the young (0.067) and middle-aged groups (0.089) but has no significant impact on that of the older group. In addition, faith (−0.069) only has a significant impact on the health of the young group. Marital status and education significantly affect the health of the young (−0.082 and 0.077, respectively) and middle-aged groups (0.127 and 0.050, respectively). Reading and exercise significantly affect the health of the young (−0.084 and 0.112, respectively) and older groups (0.188 and 0.218, respectively). Smoking, family income, and village size significantly affect the health of the middle-aged (0.100, 0.085, and −0.748, respectively) and older groups (0.119, 0.063, and −0.699, respectively). Gender (0.198, 0.218, and 0.086, respectively), age (−0.028, −0.033, and −0.007, respectively), noon break (−0.048, −0.059, and −0.180, respectively), alcoholism (0.147, 0.227, and 0.342, respectively), and family size (0.028, 0.016, and 0.019, respectively) have significant impacts on health in all age groups.

#### Income

The unbalanced development of China has resulted in a serious income gap, thereby giving rise to a possible heterogeneity in the impact of the toilet revolution on residents' health in terms of family income. This section divides the sample into the low- (6,216), middle- (6,246), and high-income (6,241) groups based on net household income per capita.

Table 7 shows that access to sanitary toilets significantly influences the health of adults from low-income households but does not significantly affect that of adults from middle- and high-income households. Given that China's low-income residents have no access to advanced medical treatment, the disease prevention function of sanitary toilets is particularly important for this group. The input-output ratio for retrofitting a sanitary toilet is approximately 1:5.3, and the benefits mainly include disease prevention and improvements in health (49). Therefore, the toilet revolution can help alleviate poverty and improve well-being.

Gender, age, education, noon break, alcoholism, exercise, and family size significantly affect the self-rated health of all groups, whereas the effects of the other variables vary in significance across all groups.

## Discussion

“Toilet revolution” is a buzzword in China. Under this background, This paper examined how access to sanitary toilets affects the health of rural adult residents. Such effect was explored at the micro-individual level by using data from the CFPS 2014 and the ordered probit model. The relationship between toilet revolution and residents' health was then illustrated to solve the public health problems in developing countries that are attributable to poor sanitation.

To mitigate estimation bias, the individual, household, and village data were matched to crowd out some confounding factors. Some health indicators were replaced, and alternative samples were utilized to guarantee the robustness of the results. The findings confirm that access to sanitary toilets significantly improves residents' health, which is consistent with the conclusions of other scholars (50, 51). Therefore, governments should invest in sanitary toilets. However, given that the rural toilet revolution is a complex and dynamic system, traditional technologies and management methods need to be improved (50). Developing countries in particular have limited funds for improving their rural health systems (2, 52). Given that the market is still at a rudimentary stage, the toilet revolution is mainly funded by national subsides (53). To make up for the lack of funds, governments should attract private capital by arousing the enthusiasm of the public while increasing the transfer payment for the toilet revolution.

This study also explored the mechanism by which access to sanitary toilets may affect public health. Fecal pathogens can lead to serious infectious diseases, and the poor sanitary toilet facilities in rural areas have increased the risk of fecal contamination in their water sources (54, 55). By dividing the samples into the “tap water” and “no tap water” groups, this study revealed that the use of sanitary toilets improved public health by preventing feces from contaminating water sources, and these results are consistent with those of previous studies (34). These results also underscore the significance of investing in sanitary toilets for environmental protection (56) and suggest that advanced technologies should be used in these toilets to improve their feces collection and storage capacities and to prevent water pollution caused by feces leakage. The government should also take measures to ensure water safety in rural areas, such as by installing water filters and establishing piped water systems.

Some heterogeneity was also observed in the impact of access to sanitary toilets on health in terms of gender, age, and income, thereby suggesting that the toilet revolution should be carried out in an orderly manner. Governments should pay more attention to females, residents aged 16 to 45 years, and people from low-income households in their implementation. Gender, age, education, marital status, alcoholism, smoking, exercise, family size, and family per capita income also had significant impacts on health status.

This study has several limitations. First, given the lack of data, this study was unable to use panel data to draw conclusions, and using of cross-sectional data to verify causation may lead to biased estimates. Second, given that health in this study was measured using a single-item question, only the effect of the toilet revolution on the overall health of adults was assessed. Third, due to the CPFS questionnaire contents, flush toilets was used to represent sanitary toilets. Fourth, not all variables affecting the health were considered in this study, and the unobserved confounders could not be controlled. Future studies should then analyze the relationships among different types of sanitary toilets and indicators of health using panel data.

## Conclusions

This is the first study to analyze the impact of China's toilet revolution on rural residents' health at the micro-individual level. Results show that access to sanitary toilets in rural households significantly improve their self-rated health, respondent-rated health, and life satisfaction. Among all subgroups, the effect of toilet revolution on health is more pronounced among females, middle-aged people, and residents from low-income households. Preventing fees from contaminating water sources is also identified as the mechanism by which residents' health is improved by using sanitary toilets. These findings suggest that governments in developing countries should invest in sanitary toilets. Future research should estimate the effects of different types of sanitary toilets on various indicators of health using panel data.

On the basis of the above conclusions, several policy recommendations are proposed. First, governments should further publicize their goal of toilet renovation to improve public health awareness and encourage rural residents to take renovate their own toilets. Second, due to limited funds, targeted and orderly promotion methods should be adopted to promote the toilet revolution in a classified manner in China. Third, governments should focus on improving their toilet technologies, especially the technologies for collecting and treating feces. Fourth, to improve fund utilization efficiency, a more reasonable toilet revolution scheme should be formulated.

## Data availability statement

The datasets presented in this study can be found in online repositories. The names of the repository/repositories and accession number(s) can be found below: http://www.isss.pku.edu.cn/cfps/.

## Author contributions

BC conceived the study design, conducted the statistical analyses, and reviewed the manuscript. FJ prepared the manuscript and supervised all aspects of its implementation. YZ prepared the manuscript and provided advice on writing the article. All authors have read and agreed to the published version of the manuscript.
